# Study of the Kinetics of Radiation Damage in CeO_2_ Ceramics upon Irradiation with Heavy Ions

**DOI:** 10.3390/ma16134653

**Published:** 2023-06-28

**Authors:** Sholpan G. Giniyatova, Artem L. Kozlovskiy, Ruslan M. Rspayev, Daryn B. Borgekov, Maxim V. Zdorovets

**Affiliations:** 1 Engineering Profile Laboratory, L.N. Gumilyov Eurasian National University, Satpayev St., Astana 010008, Kazakhstan; giniyatova_sh@enu.kz (S.G.G.);; 2 Department of General Physics, Satbayev University, Almaty 050032, Kazakhstan; 3 Laboratory of Solid State Physics, The Institute of Nuclear Physics, Almaty 050032, Kazakhstan; 4 Department of Intelligent Information Technologies, Ural Federal University, 620075 Yekaterinburg, Russia

**Keywords:** radiation defects, swelling, heavy ions, fission fragments, inert matrix materials

## Abstract

In this work, the effect of irradiation with heavy Kr^15+^ and Xe^22+^ ions on the change in the structural and strength properties of CeO_2_ microstructural ceramics, which is one of the candidates for inert matrix materials for dispersed nuclear fuel, is considered. Irradiation with heavy Kr^15+^ and Xe^22+^ ions was chosen to determine the possibility of simulation of radiation damage comparable to the action of fission fragments, as well as neutron radiation, considering damage accumulation at a given depth of the near-surface layer. During the research, it was found that the main changes in the structural properties with an increase in the irradiation fluence are associated with the crystal lattice deformation distortions and the consequent radiation damage accumulation in the surface layer, and its swelling. Evaluation of the effect of gaseous swelling caused by the radiation damage accumulation showed that a variation in the ion type during irradiation results in a growth in the value of swelling and destruction of the near-surface layer with the accumulation of deformation distortions. Results of the strength variation demonstrated that the most intense decrease in the near-surface layer hardness is observed when the fluence reaches more than 10^13^–10^14^ ion/cm^2^, which is typical for the effect of overlapping radiation damage in the material.

## 1. Introduction

Interest in the study of radiation damage in nuclear structural materials is associated with the possibility to study kinetics of changes in structural, strength, and thermophysical parameters in near-surface layers, and to establish the dose dependences of the degradation of material properties [1,2]. First, these studies are aimed at establishing the relationship between changes in the properties of materials and the dose (fluence) of radiation, as well as the type of ionizing radiation and its energy [3,4]. These studies provide a large amount of experimental data on the properties of nuclear materials, the totality of which further allows us to assess how applicable these materials are in the declared field of nuclear energy, as well as their criteria for resistance to external influences [5,6]. Second, a number of experimental works have shown the kinetics of changes in the structural characteristics of radiation-resistant materials under the influence of heavy ions in real time. The authors of a number of works [7,8] managed to establish changes in the properties of materials caused by the formation of radiation-induced damage, as well as the thermal effects associated with them. These experiments make it possible to estimate the time frame for the formation of radiation damage and to establish their evolution in the damaged material layer [7,8]. Third, the study of radiation damage in new types of nuclear materials, in particular oxide, nitride, and carbide ceramics, makes it possible to obtain new data on the properties of these materials, which have become increasingly interesting in recent years [9,10,11]. At the same time, interest in inert matrices is not only due to their high mechanical and thermophysical parameters, but also to the variety of ways to obtain them. The most common method for obtaining various composite ceramics based on binary or ternary compounds is the method of mechanochemical solid-phase synthesis, the use of which makes it possible to vary the phase composition, as well as the size of the obtained grains by changing the grinding conditions and subsequent thermal annealing [12]. Another common production method is thermal or plasma spraying, however, in this case, there are a number of limitations related to the size of the grains obtained, as well as the control of structural characteristics, etc. [13]. Moreover, sometimes the sol-gel method is used as a method for obtaining ceramics, which includes chemical reactions with the formation of colloidal solutions, which are subsequently subjected to heat treatment and sintering of samples [14].

Among the studies aimed at establishing the resistance of nuclear structural materials to radiation damage, a special role is occupied by experiments aimed at the study of the processes of radiation-induced changes in dispersed nuclear fuel materials [15,16]. Interest in this class of materials is primarily owing to the possibility of creating alternative types of nuclear fuel, the concept of which is to increase the degree of burnup of nuclear fuel, as well as the possibility of improving operating conditions with the transition to high-temperature nuclear reactors [17,18]. The use of inert matrices of dispersed nuclear fuel as an alternative type to traditional fuel elements will increase the percentage of fissile material burnup, as well as switch to the use of plutonium as the basis for fissile material, the use of which will reduce the amount of nuclear waste generated [19,20,21]. In this regard, the study of the processes of radiation-induced damage in inert matrices, by modeling radiation damage comparable to fission fragments of fissile nuclear fuel, will allow us to assess the prospects for using ceramics as the basis of dispersed nuclear fuel. Among the variety and many different options and types of ceramic materials, cerium dioxide should be singled out [22,23], which has rather great prospects for using dispersed nuclear fuel as inert matrices due to its physical and chemical properties (absence of polymorphic transformations), high strength and hardness (240–400 HV), high melting point (2400–2600 °C), good thermal conductivity (0.3–5 W m^−1^ K^−1^), as well as chemical inertness to most acids and alkalis. Moreover, high resistance to thermal effects makes cerium dioxide-based ceramics one of the promising materials as inert matrices for dispersed nuclear fuel, as well as as a basis for dielectric or insulating composites [24,25,26].

The key aim of this study is to investigate mechanisms of accumulation of radiation damage and associated deformation distortions in the near-surface layer, leading to softening and destruction of the ceramic material when it comes to high-dose irradiation with heavy Kr^15+^ and Xe^22+^ ions. Interest in these studies is mainly on account of the opportunity to simulate processes associated with the radiation damage accumulation in promising nuclear materials, and to study the consequences of the accumulation of radiation-induced structural changes in the near-surface layer, which is most susceptible to external influences, mechanical pressure, and aggressive media during operation. At the same time, experiments related to irradiation with heavy Kr^15+^ and Xe^22+^ ions make it possible to create the most realistic conditions for the radiation damage occurrence in inert matrices during their operation in the event of using materials as inert matrices of dispersed nuclear fuel [27,28].

## 2. Experimental Part

**Figure 1:**
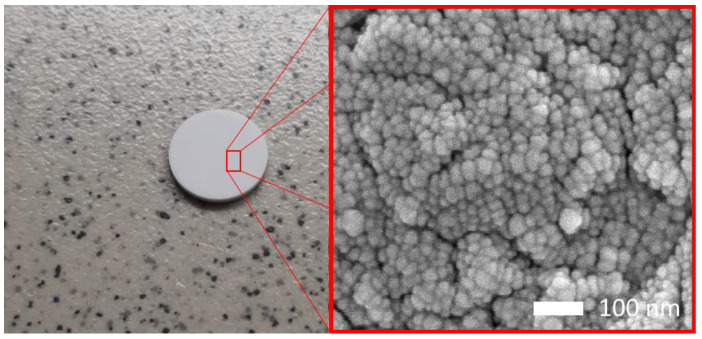
Presentation results of the obtained CeO_2_ ceramics.

Ceramic samples based on cerium dioxide, obtained by pressing in a special mold with a diameter of 10 mm and 1 mm, were chosen as initial samples. The samples were pressed from nanostructured powders of cerium dioxide (CeO_2_) with a chemical purity of 99.95%, manufactured by Sigma Aldrich (Sigma, St. Louis, MI, USA). Figure 1 illustrates typical images of the obtained ceramics after pressing, and the inset shows an image of the morphological features of the ceramics, made using scanning electron microscopy. The images were taken using a Hitachi TM3030 scanning electron microscope (Hitachi, Tokyo, Japan) in the LEI mode, with an accelerating voltage of 15 kV. Ceramic samples in the form of tablets were obtained by pressing in a special mold at a pressure of 300 MPa, the pressure was maintained for 30 min, after which the samples were removed and annealed in a Nabertherm muffle furnace (Nabertherm, Lilienthal, Germany) at a temperature of 1000 °C for 20 h in order to anneal defects and mechanical stresses that arose during pressing. The porosity of the samples did not exceed 1–2% (determined by the Archimedes method), the density of the samples according to the Archimedes method was 7.59 g/cm^2^.

As can be seen from the provided data on the study of morphological features, the surface of ceramics is a close-packed finely dispersed (less than 70 nm) grain structure. The presence of small microcracks along the boundaries of grain agglomerates is associated with the processes of polishing ceramics, as well as the processes of their formation.

The study of the radiation resistance of CeO_2_ ceramics, along with the study of the radiation-induced swelling and embrittlement mechanisms, was carried out by simulation of the processes of irradiation with heavy Kr^15+^ and Xe^22+^ ions at the DC-60 accelerator (Institute of Nuclear Physics, Almaty, Kazakhstan). The energies of the Kr^15+^ and Xe^22+^ ions were 147 and 230 MeV, respectively; the irradiation fluences were chosen from 10^10^ to 10^14^ ion/cm^2^. Irradiation was executed at a temperature of 1000 K in order to simulate the conditions of accumulation of radiation damage in ceramics as close as possible to real operating conditions (close to the conditions of the core with nuclear fuel for high-temperature reactors). The choice of irradiation fluences is due to the possibility of modeling the processes of formation of radiation-induced damage in ceramics both in the case of the formation of single damaged areas that occur during the passage of incident ions in the material at low fluences, and in the case of overlapping of these areas in the case of high-dose irradiation.

**Figure 2:**
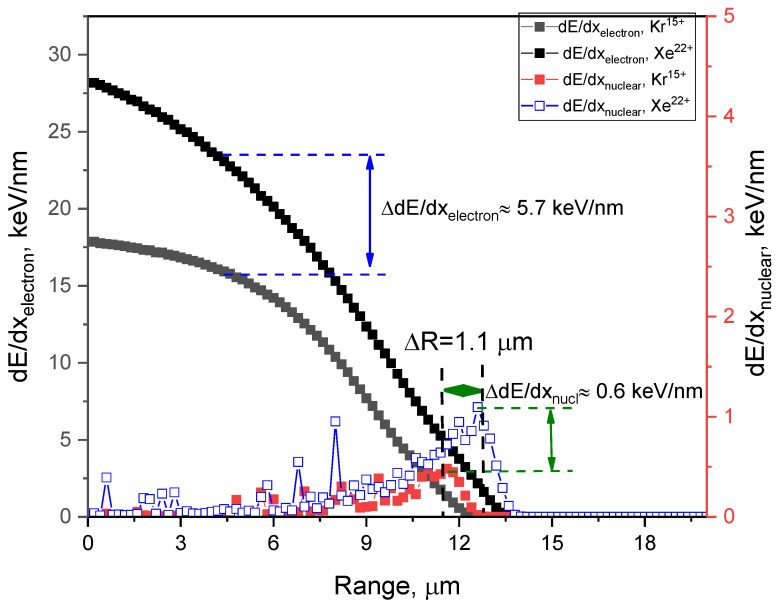
Calculation data for the ionization losses of incident ions of Kr^15+^ and Xe^22+^ ions with energies of 147 and 230 MeV in a CeO_2_ ceramics target.

Figure 2 demonstrates the assessment results of the energy losses of incident Kr^15+^ and Xe^22+^ ions with energies of 147 and 230 MeV, that arise during collisions with the crystal structure of the target. These calculations were performed using the SRIM Pro 2013 program code; the Kinchin–Pease model was used in the simulation, taking into account cascade collisions and their contribution to the change in ion energy losses. As can be seen from the presented data, the maximum range of the Kr^15+^ and Xe^22+^ ions in CeO_2_ ceramics is 12.1 and 13.2 µm, respectively, with the difference in range being no more than 1.1 µm. At the same time, significant differences are observed in the energy losses of incident ions during interaction with electron shells. In this case, the maximum value of dE/dx_electron_ is no more than 17.5 keV/nm and 23.2 keV/nm for Kr^15+^ and Xe^22+^ ions, respectively, which indicates a greater influence of electron losses and the consequences associated with ionization processes and the formation of cascade effects during irradiation with heavy ions Xe^22+^. As can be seen from the data presented, when the type of ions changes during irradiation from Kr^15+^ to Xe^22+^, the change in the value of nuclear losses is no more than 0.6 keV/nm. At the same time, it should be noted that the main contribution to the change in the structural properties of the material is made by the electron losses of incident ions over most of the path length, since their value is two orders of magnitude higher. At the same time, nuclear losses play a key role in the case of reaching the maximum range of ions, when the stopping power of ions is based on a large number of nuclear collisions. In this case, nuclear losses grow and lead to an increase in atomic displacements caused by irradiation.

**Figure 3:**
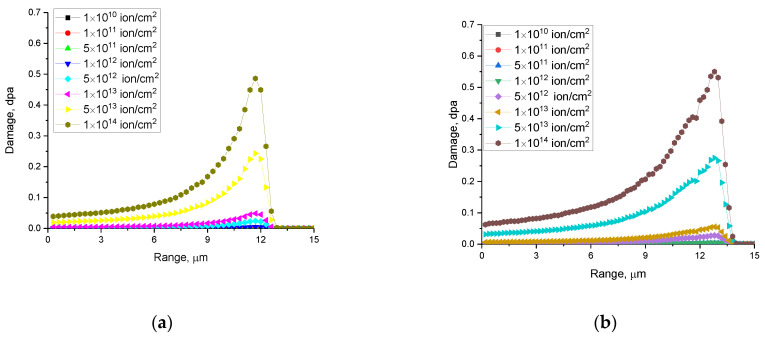
Evaluation results of alterations in the values of atomic displacements along the trajectory of ions in the material under irradiation with Kr^15+^ (**a**) and Xe^22+^ (**b**) ions.

Using the method for calculating the atomic displacements in a damaged layer of ceramics caused by irradiation with heavy Kr^15+^ and Xe^22+^ ions, proposed in [29], dependences of the atomic displacements along the trajectory of ions in the material were calculated. The calculation results are shown in Figure 3.

As evident from the data provided, the maximum possible achievement of atomic displacements in the case of irradiation with heavy Kr^15+^ and Xe^22+^ ions at a fluence of 10^14^ ion/cm^2^ is 0.48 and 0.55 dpa, respectively. Moreover, the difference in the magnitude of atomic displacements when the type of incident ions changes from Kr^15+^ and Xe^22+^ is no more than 10%, while the energy of incident ions changes by 80 MeV. Therefore, analyzing the data on the values of atomic displacements and ionization losses, we can conclude that the main effect on the structural changes in ceramics will be the processes of interaction of incident ions with electron shells, accompanied by ionization processes and changes in the electron density distribution and the formation of vacancy defects.

The presented results of the accumulation of atomic displacements in the damaged layer are in good agreement with the results of [28], which describes the latest studies of radiation damage in CeO_2_ ceramics. It should be noted that the small value of atomic displacements is due to the fact that at given energies of incident ions 147 and 230 MeV, as well as irradiation fluences 10^10^ to 10^14^ ion/cm^2^, the main contribution to the processes of defect formation is made by the effects associated with the loss of ions during interaction with electron shells, leading to ionization and the formation of structural distortions of the crystal lattice due to the formation of vacancy and point defects, as well as residual stresses.

The study of the resistance of CeO_2_ ceramic materials to radiation-induced changes in structural features associated with deformation distortions and partial disordering of the near-surface damaged layer was conducted through the method of X-ray diffraction analysis. The measurements were performed using a D8 Advance ECO X-ray diffractometer (Bruker, Berlin, Germany). X-ray diffraction patterns were taken in the Bragg–Brentano geometry in the angular range 2θ = 20–90°, with a step of 0.03°. Structural parameters were evaluated using the DiffracEVA v.4.2 program code. To calculate the structural distortions, as well as the formation of structurally disordered regions in the near-surface damaged layer caused by irradiation, we used assumptions about the influence of various factors on the change in the shape, intensity, and magnitude of the FWHM of diffraction reflections. All measurements of the structural features were performed taking into account the depth of the path of the incident ions by selecting the optimal conditions for taking X-ray diffraction patterns in a way that the resulting diffraction patterns cover the irradiated region in the entire irradiated volume.

The value of amorphous inclusions or structurally disordered regions arising as a result of deformation distortions and stresses during the radiation damage accumulation was calculated based on the data on changes in the ratio of the areas of diffraction reflections and background radiation, which characterizes the presence of disorder in the composition of the studied samples. This value is an average value characterizing the structural disorder in the composition of ceramics associated with changes caused by irradiation and the subsequent kinetics of radiation-induced damage. An estimate of the swelling of the crystal lattice, as well as its deformational distortion, was calculated based on the data on changes in the parameters of the crystal lattice in comparison with the values of the original sample. To avoid the effects of residual deformation distortions in the ceramic samples after being pressed into pellets, the samples were subjected to thermal annealing for 20 h at a temperature of 1000 °C in a muffle furnace. The samples were annealed in an air atmosphere in a muffle furnace.

Tests for determining the strength of ceramics were carried out using the indentation method implemented on a LECO LM700 microhardness tester (LECO, Tokyo, Japan). The hardness values were determined using a Vickers diamond pyramid, with a load value of 1 kN. To determine the accuracy of measurements and the error of values, all tests were performed serially in 15–20 consecutive tests to determine the values of hardness and geometry of the resulting indenter prints. At the same time, the measurements were performed at more than 10 microns from each other in order to avoid the effects of cracking and deformation distortions near the prints.

## 3. Results and Discussion

**Figure 4:**
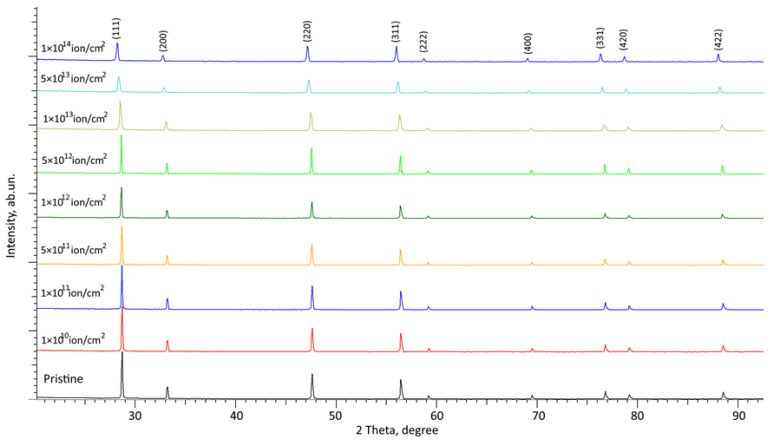
Results of X-ray diffraction of the investigated CeO_2_ ceramics subjected to irradiation with Kr^15+^ ions.

**Figure 5:**
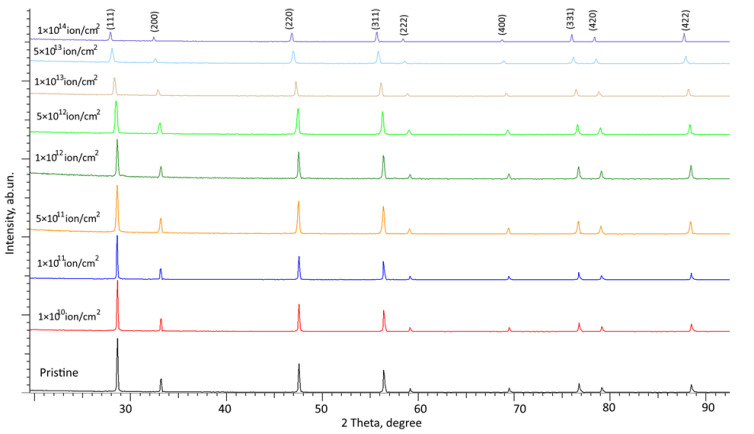
Results of X-ray diffraction of the studied CeO_2_ ceramics subjected to irradiation with Xe ^22+^ ions.

Figure 4 and Figure 5 show the results of X-ray diffraction of the studied CeO_2_ ceramic samples depending on the fluence of irradiation with heavy Kr^15+^ and Xe^22+^ ions, reflecting the dynamics of changes in the structural features of the samples as a result of radiation damage accumulation. The position of the main observed diffraction reflections, and the ratio of their intensity, is typical for CeO_2_ structures with a face-centered crystal lattice, with a parameter of 5.3916 Å, slightly different from the reference value (a = 5.4116 Å), corresponding to the PDF-01-070-8371 card value. This difference is associated with the manufacturing processes of ceramics, along with their subsequent thermal annealing, which is applied to relax deformation distortions, the absence of which is evidenced by the symmetrical shape of diffraction reflections.

The overall appearance of the observed changes suggests that in both cases, irradiation with heavy Kr^15+^ and Xe^22+^ ions, even at maximum fluences, does not result in processes associated with phase or polymorphic transformations, and the main observed changes are primarily caused by the mechanisms of structural disorder and accumulation of deformation distortions of the crystal structure. The absence of new diffraction reflections in the obtained diffraction patterns suggests that during irradiation there are no phase transformations in the structure of CeO_2_ ceramics, which confirms the high resistance of this class of ceramics to phase transformations and processes associated with the formation of new phase inclusions caused by thermal effects and deformation distortions. At the same time, the overall picture of the presented changes in X-ray diffraction patterns depending on the irradiation fluence indicates the occurrence of two main processes of structural changes associated with deformation distortion and a change in the intensity and FWHM of diffraction lines characteristic of amorphization processes. An analysis of the change in the shape of the diffraction lines, their displacement relative to the initial position in comparison with the initial samples, indicates the processes of deformation distortion caused by the radiation damage accumulation in the damaged layer. At the same time, a drop in the intensity of diffraction reflections observed with a growth in irradiation fluences above 10^12^ ion/cm^2^ indicates not only deformation distortions, but also processes of partial amorphization or the formation of structurally disordered regions in the damaged near-surface layer of ceramics.

According to [30,31], upon irradiation with Xe^14+^ [30] heavy ions with an energy of 70–210 MeV and Xe^23+^ with an energy of 92 MeV [31], the formation of structurally disordered regions, i.e., latent tracks, is observed, the visualization of which occurs during subsequent chemical treatment. The reasons for the formation of latent tracks, as well as changes in their sizes, are associated by the authors with a possible change in the ratio of Ce^3+^ and Ce^4+^ ions near the surface of thin films, as well as a change in the charge balance in films, the change which is evidenced by a change in the electrical conductivity of thin films [31]. The authors of [30] also established the dependence of latent track diameters in CeO_2_ on the irradiation temperature and energy of incident ions, according to which an increase in the irradiation temperature leads to a decrease in track diameters, while an increase in the energy of incident ions leads to an increase in diameters. At the same time, the nature of latent tracks, according to the works [32,33], has a pronounced dependence on the type of ions, as well as their charge state, which in the case of dielectric materials leads to large changes in the electron density, as well as the formation of an anisotropic distortion. At the same time, in the works [30,31], as in the case of this work, irradiation with heavy ions does not lead to phase transformations and polymorphic transformations, and the mechanisms of formation of latent tracks, according to the works [30,31,32,33], are accompanied by the formation of defective inclusions and residual stresses associated with deformation distortion of the crystal structure of the damaged layer.

**Figure 6:**
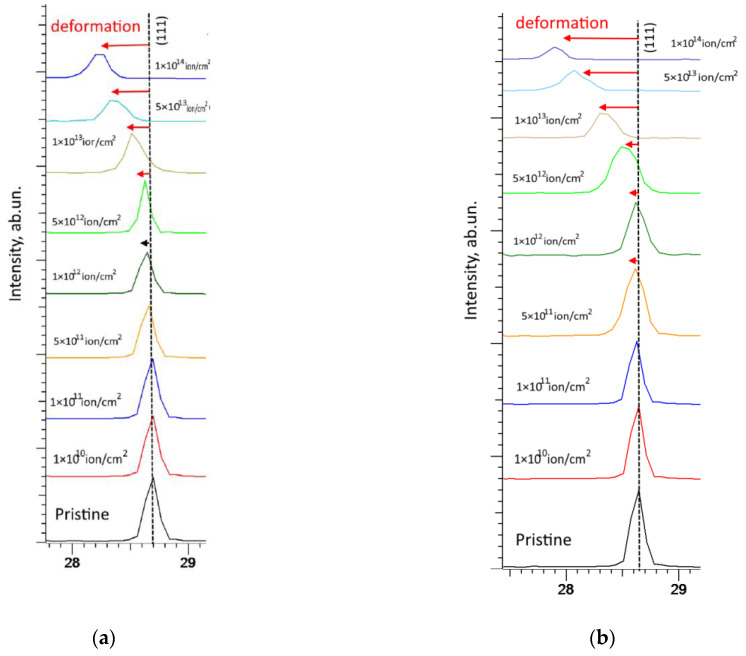
Detailed image of distortion and deformation displacement of the (111) reflection upon irradiation with Kr^15+^ (**a**) and Xe^22+^ (**b**) ions.

Figure 6 illustrates comparative data on alterations in the shape and position of the main (most intense) diffraction reflection (111), which characterizes the main textural direction in a face-centered crystal lattice. These changes in position and diffraction intensity characterize the main types of structural changes caused by irradiation.

**Figure 7:**
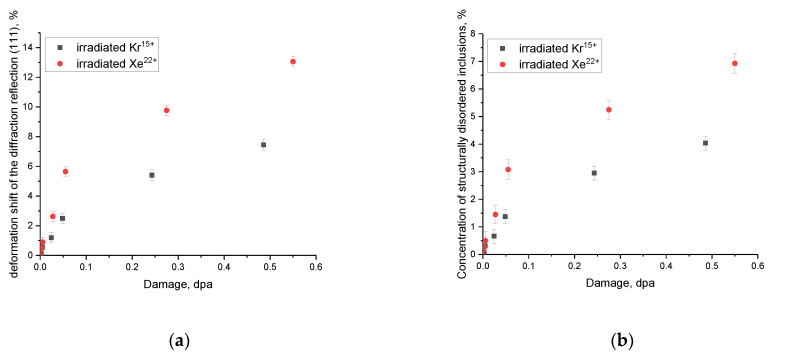
(**a**) Results of the deformation displacement of the diffraction reflection (111) as a function of the value of atomic displacements upon irradiation with heavy ions; (**b**) results of evaluation of the contributions of formed structurally disordered inclusions to the near-surface damaged layer of CeO_2_ ceramics.

As evident from the presented comparative data, variations in the position and shape of the (111) diffraction reflection depending on the type of ions and the irradiation fluence, the most pronounced changes are observed at fluences above 10^11^ ions/cm^2^. When it comes to irradiation with Xe^22+^ ions, these changes appear at a fluence of 5 × 10^11^ ion/cm^2^, while upon irradiation with Kr^15+^ ions, a shift of reflections is observed at a fluence of 10^12^ ion/cm^2^. The shift of the position of the maximum of the diffraction reflection relative to the initial value (marked by a dotted line in the figure) to the region of small angles indicates the presence of tensile type deformation distortions, which are formed as a result of the accumulation of radiation damage, vacancy, and point defects. At irradiation fluences above 5 × 10^12^ ion/cm^2^, a decline in intensity is observed along with a strong broadening of the reflections, indicating a rise in the FWHM value. At the same time, in the case of irradiation with Xe^22+^ ions, these changes become more pronounced under high-dose irradiation, which indicates a higher concentration of radiation-induced damage in the near-surface layer caused by irradiation. On the basis of these changes, the deformation factor of distortion of the diffraction reflection (111) was calculated, the data of which are presented in Figure 7a. As can be seen from the data presented, a rise in the irradiation fluence (an increase in the magnitude of atomic displacements) results in a growth in deformation distortion and a shift of diffraction reflections to the region of small angles, which indicates the tensile nature of the deformation of the crystal structure. Moreover, in the case of irradiation with Xe^22+^ heavy ions, the deformation distortions are almost 1.5–2 times higher than in the case of irradiation with Kr^15+^ ions. At the same time, the trend of changes in deformation distortions has a clear decrease with an increase in the irradiation fluence, which may be due to the effects of accumulation and a decrease in the migration rate of defects during high-dose irradiation. This decrease in the migration rate can be explained by an increase in deformation inclusions that prevent the migration of defects, thereby forming agglomerates or highly defective areas. It should also be noted that this significant difference in the structural distortion values for different ions may be due to the fact that in the case of irradiation with Xe^22+^ heavy ions, the region subjected to structural distortion along the ion motion trajectory is considerably larger, and high values of ionization losses in the collision of incident particles with the crystal structure result in the formation of a large number of point and vacancy defects [3,4]. Therefore, high ionization losses of incident Xe^22+^ ions can initiate a greater number of structural distortions, which, at high irradiation fluences, can lead to accelerated amorphization and degradation, as shown in several studies on the irradiation of nitride ceramics [32,34].

Figure 7b reveals the assessment results of the contribution of structurally disordered (amorphous) inclusions to the damaged near-surface layer of ceramics depending on the fluence of irradiation with different types of ions. These calculations were performed by estimating the areas of diffraction reflections and background radiation, which characterizes the structural disorder of the crystal structure caused by external influences. As can be seen from the data provided, the development of amorphous inclusions occurs in the case of overlapping of defective areas in the near-surface damaged layer characteristic of high-dose irradiation (above 10^12^ ion/cm^2^), resulting in a destructive change in the properties of ceramics and can have a negative impact on resistance to external influences. The formation of structurally disordered inclusions occurs as a result of the cumulative effect, which is due to deformation distortions and stresses caused by irradiation. At the same time, according to the assessment of ionization losses, as well as their comparison with the caused structural changes, it can be concluded that electronic losses have a great influence on deformation distortions and the formation of structurally disordered regions. This is primarily due to ionization effects associated with a change in the electron density, as well as the formation of vacancy and point defects, which, migrating in the damaged layer, lead to its destabilization and the appearance of deformation distortions and stresses.

It was found that in the case of high-dose irradiation with heavy ions, this effect is cumulative, indicating the formation and subsequent accumulation of radiation damage, their agglomeration, and the occurrence of structural distortions in the crystal structure and its disordering. The formation of amorphous inclusions and regions of disorder occurs at maximum irradiation fluences and amounts to no more than 4–7%, which indicates a rather high resistance of ceramics to radiation disorder.

**Figure 8:**
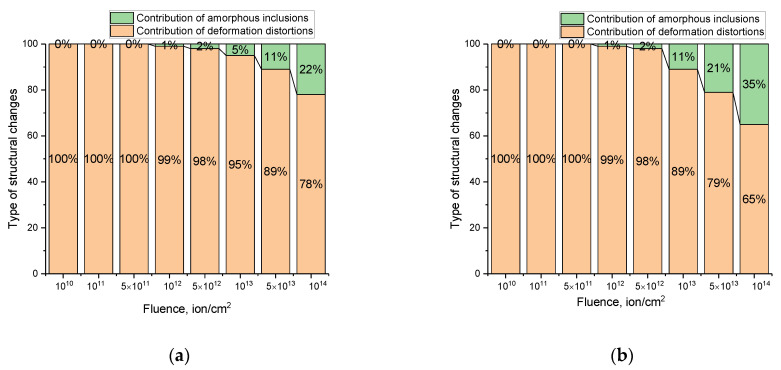
Results of evaluation of the contributions of deformation distortions and the formation of amorphous or structurally disordered inclusions to the change in the properties of the near-surface CeO_2_ layer of ceramics upon irradiation with heavy Kr^15+^ (**a**) and Xe^22+^ (**b**) ions.

Figure 8 shows the results of evaluating two contributions to the change in the diffraction pattern of ceramic samples subjected to irradiation. Deformation distortions associated with structural deformations of the crystal lattice and the concentration of amorphous inclusions resulting from the radiation damage accumulation were considered as two contributions. To determine the contributions, it was assumed that in the initial state (in the unirradiated sample), there are no structural deformations and amorphous inclusions. In the case of irradiated samples, any change in the shape, intensity, and position of diffraction reflections characterizes the presence of deformation distortions or amorphous inclusions associated with the effect of crystal lattice irradiation. At the same time, the contribution of deformation distortions was estimated from the value of the shifts in the position of diffraction maxima, and the contribution of amorphous inclusions was determined from the ratio of the areas of diffraction reflections and background radiation.

As can be seen from the data presented in Figure 8, the main contribution to the change in the properties of ceramics at irradiation fluences of 10^10^–10^12^ ion/cm^2^ is made by deformation distortions of the crystal lattice due to tensile stresses formed in the near-surface damaged layer. In this case, the deformation distortion is primarily due to ionization processes associated with the passage of heavy ions in ceramics, followed by excitation of the crystal lattice and a change in its thermal vibrations. At the same time, high-temperature irradiation in the case of low irradiation fluences (10^10^–10^12^ ion/cm^2^) can annihilate the effect associated with structural disorder and the formation of amorphous inclusions due to a change in thermal vibrations of the crystal lattice. In the case of the formation of overlapping defective regions at irradiation fluences above 10^12^ ion/cm^2^, thermal vibrations during high-temperature irradiation are unable to reduce the effect of structural disorder, which leads to the formation of amorphous inclusions in the structure of ceramics. The formation of amorphous inclusions was determined from the analysis of the shape, intensity, and value of the FWHM diffraction lines, taking into account the Debye–Waller factors, according to which, a change in the crystal lattice volume and density implies amorphization of the structure. When it comes to irradiation with Xe^22+^ ions, the contribution of amorphous inclusions at fluences above 10^13^ ion/cm^2^ is significantly larger than for samples irradiated with Kr^15+^ ions. This difference can be primarily due to the fact that in the case of irradiation with Xe^22+^ ions, the characteristic sizes of defect regions that appear along the ion motion trajectory in the material are considerably larger than in the case of irradiation with Kr^15+^ ions. The result of these differences may be an increase in the effect of overlapping of these areas, which introduces more structural distortions, leading in the case of overlap and high concentration to partial amorphization of the damaged layer. As shown in [32], with an increase in the energy of incident ions, as well as a change in their type, the destructive processes associated with amorphization caused by the accumulation of residual stresses in the damaged layer occur significantly faster and at lower fluences. The authors of [32] attribute this effect to a change in the diameters of damaged regions (latent tracks) that appear along the trajectory of incident ions in the material, as well as to large energy losses when the type of incident ions and their energy change. The results obtained in [32] are a direct confirmation of the influence of the type of incident ions on the processes of defect formation and their accumulation in the structure of the damaged layer.

One of the factors affecting the change in the properties of ceramics due to deformation distortions is the assessment of the nature of the isotropy of these changes, as well as the occurrence of textural effects associated with greater or lesser resistance to structural changes. The isotropy of structural distortions associated with texture effects, along with the reorientation of crystallites during irradiation, can be estimated by studying the dynamics of changes in texture coefficients that reflect the priority orientation of crystallites and texture planes. At the same time, the analysis of changes in texture coefficients depending on the conditions of external influences can reflect the crystallite reorientation effects, as well as recrystallization processes.

**Figure 9:**
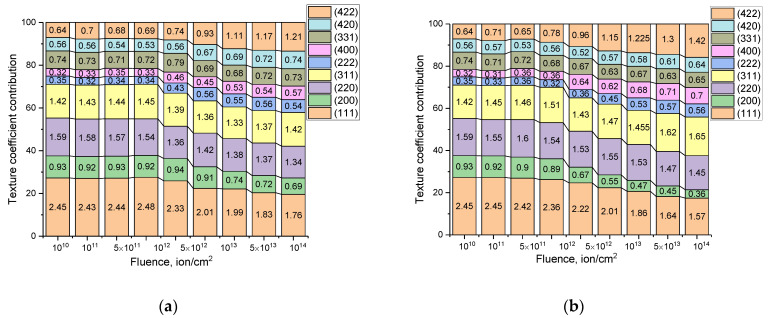
Results of evaluation of alterations in texture coefficients depending on the fluence of irradiation with Kr^15+^ (**a**) and Xe^22+^ (**b**) ions.

Based on the data on changes in texture coefficients for the studied polycrystalline ceramics, the influence of irradiation with heavy ions on the effects of crystallite reorientation as a result of irradiation, as well as the processes of texture change as a result of external influences, was studied. Figure 9 shows the results of estimating the contributions of changes in texture coefficients calculated on the basis of the intensity of diffraction reflections and their contributions. The texture coefficients were calculated using the methods proposed in [35,36].

As can be seen from the presented data, the most pronounced changes in texture coefficients are observed at fluences above 10^12^ ion/cm^2^, which consist of a decrease in the contribution of the main texture direction (111) with an equiprobable increase in all other texture coefficients. These changes indicate an equiprobable distribution of distorting deformation inclusions in the structure, without significant processes of reorientation of crystallites as a result of external influences. The decrease under high-dose irradiation is due to the processes of amorphization and destruction of the near-surface layer associated with the accumulation of deformation distortions.

**Figure 10:**
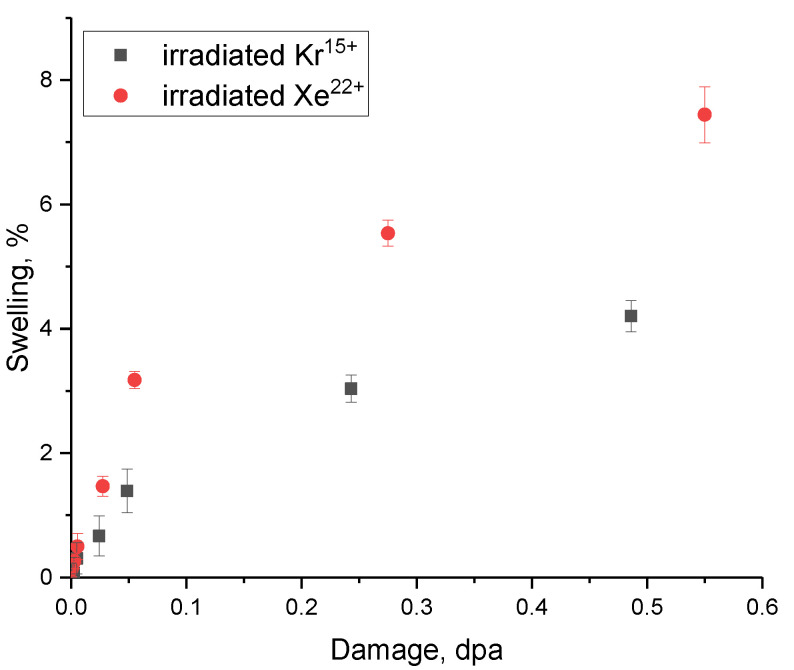
Results of crystal lattice swelling as a function of the accumulated radiation damage in the structure.

Figure 10 demonstrates the assessment results of the crystal lattice swelling value associated with a change in its volume due to the accumulation of radiation damage, deformation distortions, and amorphous inclusions. As evident from the presented data, the most pronounced effects of crystal lattice swelling as a result of the structural distortion accumulation, along with the possibility of filling voids formed in the crystal structure with implanted Kr^15+^ and Xe^22+^ ions, are observed at fluences above 10^12^ ion/cm^2^ (at atomic displacements above 0.01 dpa). At the same time, these effects are most pronounced for samples irradiated with Xe^22+^ ions, for which the swelling at the maximum irradiation fluence is more than 6%, while the variation in the crystal structure volume upon irradiation with Kr^15+^ ions is no more than 4%.

**Figure 11:**
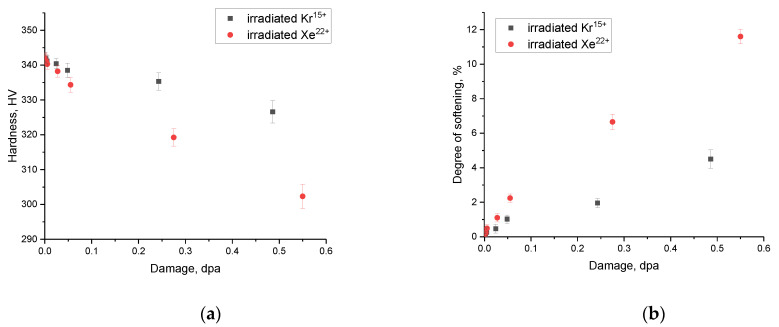
(**a**) Results of changes in the strength of ceramics as a result of irradiation; (**b**) data on the dependence of the change in the softening value of the near-surface layer of ceramics as a result of the accumulation of radiation damage.

Figure 11 shows evaluation results of strength properties of ceramics, the alteration which indicates the softening effect during the radiation damage accumulation, as well as a decline in resistance to destruction under external influences.

The data presented in Figure 11 reflect the change in the hardness of ceramics depending on the magnitude of atomic displacements caused by irradiation with heavy ions, as well as a decrease in the resistance to softening associated with a change in the hardness of the irradiated samples compared to the initial value. The physical meaning of the presented results is to reflect the dynamics of weakening and decrease in the hardness of ceramics during the accumulation of radiation damage in them, and as a result, deformation distortions leading to a deterioration in strength properties. Moreover, according to the data obtained, it can be seen that the dynamics of changes in strength properties have a pronounced dependence on the amount of accumulation of atomic displacements, at a small value of which ceramics remain resistant to softening. The overall appearance of the presented dependences suggests that the most pronounced changes in the strength properties of ceramics are observed during the radiation damage accumulation in the case of irradiation with Xe^22+^ ions. Moreover, the maximum softening of the near-surface layer of ceramics is more than 4% for samples irradiated with Kr^15+^ ions and 10% for samples irradiated with Xe^22+^ ions at the maximum irradiation fluence. These differences in the changes in strength characteristics can be due to the effects of accumulation of amorphous inclusions and more intense swelling of the crystal structure of ceramic samples upon irradiation with heavy Xe^22+^ ions. In the case of samples irradiated with Kr^15+^ ions, the maximum achievable change in the strength properties of ceramics as a result of the radiation damage accumulation is no more than 4%, which is 2.5 times less than the changes in the strength properties of ceramics irradiated with Xe^22+^ ions.

## 4. Conclusions

The paper presents the results of an assessment of radiation damage in CeO_2_ ceramics—promising materials for the creation of inert matrices of nuclear fuel, as well as having potential in the field of structural materials for new generation reactors. The choice of irradiation conditions is due to the possibilities of radiation damage simulation, as well as the processes of gas swelling of the near-surface layer during the accumulation of implanted heavy ions in the case of high-dose irradiation.

During the studies, it was found that at irradiation fluences of 10^10^–10^12^ ion/cm^2^, the main structural changes are associated with deformation distortion of the damaged layer associated with the accumulation of tensile stresses caused by interaction of incident ions with the ceramic structure. At fluences above 10^12^ ion/cm^2^, the variation in the structural characteristics of ceramics is due to deformation distortions, as well as the formation of structurally disordered (amorphous) inclusions, the concentration of which is more than 4–7% at the maximum irradiation fluence.

An analysis of alterations in the strength dependences of the studied ceramics revealed that the obtained samples have higher resistance to destructive changes in strength characteristics in the case of irradiation with Kr^15+^ ions than with irradiation with Xe^22+^ ions. At the same time, the difference in the softening indices at the maximum irradiation fluence is more than 2.5 times. Moreover, during determination of the strength properties, it was established that the greatest influence on softening is exerted by the effects associated with the formation of amorphous inclusions and crystal lattice swelling. In the case of fluences of 5×10^13^–10^14^ ion/cm^2^, which are characterized by an increase in the accumulation of atomic displacements, softening is associated with an increase in the contribution of atomic displacements and the structural changes caused by them in ceramics. In this case, an increase in atomic displacements leads to an acceleration of the destruction of ceramics, which should be taken into account in the future when designing fuels using these types of ceramics.

## Data Availability

Not applicable.
